# Association of thigh muscle status and longitudinal changes assessed by MRI with subsequent total hip arthroplasty: insights from the osteoarthritis initiative

**DOI:** 10.1186/s42836-026-00423-5

**Published:** 2026-09-01

**Authors:** Xiangjie Yin, Ziquan Li, Yuanpeng Zhu, Di Liu, Terry Jianguo Zhang, Siyi Cai, Nan Wu

**Affiliations:** 1 Department of Orthopedic Surgery, Peking Union Medical College Hospital, Chinese Academy of Medical Sciences & Peking Union Medical College, Beijing, 100730 China; 2 Beijing Key Laboratory of Big Data Innovation and Application for Skeletal Health Care, Beijing, 100730 China; 3 Key Laboratory of Big Data for Spinal Deformities, Chinese Academy of Medical Sciences, Beijing, 100730 China

**Keywords:** Osteoarthritis Initiative, Hip osteoarthritis, Total hip arthroplasty, MRI, Thigh muscle, Cross-sectional area, Body composition, Landmark analysis

## Abstract

**Background:**

Evidence regarding the association of longitudinal thigh muscle MRI markers with subsequent total hip arthroplasty (THA) is limited. Such markers may help characterize risk among individuals with or at risk of hip osteoarthritis.

**Methods:**

A longitudinal year-2 landmark analysis was conducted using the Osteoarthritis Initiative cohort. Hips that underwent THA after the year-2 landmark were matched to eligible control hips at a 1:4 ratio. Thigh MRI cross-sectional areas (CSAs) were automatically quantified using a validated U-Net segmentation network. The prespecified primary exposure was the 2-year change in total thigh muscle CSA. Individual muscle regions, adipose-tissue measures, baseline measurements, and alternative processing methods were evaluated as secondary or exploratory exposures. Cox proportional hazards models reported hazard ratios (HRs) per 1-standard-deviation increase. Secondary *p*-values were adjusted using the Benjamini–Hochberg false discovery rate procedure. This study evaluated associations and did not develop or validate a clinical prediction model.

**Results:**

A total of 495 hips (99 THA cases and 396 matched controls) were included. In the primary analysis, a greater 2-year change in total thigh muscle CSA was associated with a lower incidence of subsequent THA (HR per 1-SD increase, 0.75; 95% CI 0.63–0.90; *p* = 0.002). In secondary analyses, the 2-year changes in quadriceps, hamstrings, and adductors CSA showed nominal inverse associations with subsequent THA; however, none remained statistically significant after FDR correction (all *q* ≥ 0.05). No baseline MRI measure was significantly associated with subsequent THA.

**Conclusion:**

A greater 2-year change in total thigh muscle CSA, corresponding to greater gain or less loss, was associated with a lower incidence of subsequent THA. Conversely, lower change values or greater CSA loss were associated with higher subsequent THA incidence. Findings for individual muscle regions and alternative processing methods did not remain significant after false discovery rate correction and should be interpreted as exploratory. Longitudinal total thigh muscle CSA may warrant further study as a potential risk-stratification marker, but predictive performance and clinical utility were not evaluated.

**Trial registration:**

The parent OAI cohort is registered at ClinicalTrials.gov (NCT00080171). This secondary analysis was not separately registered.

**Supplementary Information:**

The online version contains supplementary material available at https://doi.org/10.1186/s42836-026-00423-5.

## Introduction

While total hip arthroplasty (THA) remains the definitive intervention for advanced hip osteoarthritis (OA), its increasing utilization underscores the need for improved risk assessment and preventive strategies [1]. Recent projections estimate a 71% increase in THA demand among U.S. adults by 2030 compared with 2014 (from 370,800 to 635,000 annual procedures), with the growth rate having decelerated since 2008 [2]. The substantial economic burden of THA has increased clinical interest in preclinical risk stratification in hip OA [3].

Thigh muscles play a crucial role in the biomechanical stability of the hip joint[4]. Their strength, cross-sectional area (CSA), and composition can be improved through interventions such as specific training protocols and nutritional supplementation [5–7]. Previous studies have suggested that the condition of the thigh fat and muscles may be a significant factor in hip OA. Bieler et al. underscored that thigh muscle power is crucial for identifying functional deficits in hip OA [8]. At the same time, Weng et al. demonstrated that elevated thigh intramuscular fat infiltration is significantly associated with increased incidence of both knee and hip OA [9].

However, certain previous studies are constrained by their cross-sectional designs, posing challenges in establishing causality [8]. Furthermore, numerous studies employ thigh muscle strength as a proxy for the status of thigh muscles, yet this approach is susceptible to errors and may not fully or precisely reflect the detailed status of thigh muscles [10, 11]. Thus, there is an urgent need to explore more reliable and quantitative assessment methods for thigh muscles. Quantitative markers of muscle composition assessed by MRI have been proven to be associated with clinical muscle strength in various conditions [12–14]. Currently, there have been no studies investigating the diverse components of thigh muscle composition—such as muscle mass, fat content, and their ratios—and their relationship with outcomes of hip OA, such as progression or the incidence of future THA.

Therefore, we conducted a longitudinal year-2 landmark analysis using the Osteoarthritis Initiative (OAI), a prospective observational cohort of individuals with or at risk of OA. We prespecified the 2-year change in total thigh muscle CSA as the primary exposure and evaluated individual muscle regions, adipose-tissue measures, baseline measurements, and alternative processing methods as secondary or exploratory exposures in relation to subsequent THA.

## Methods

### Study sample and participants

This longitudinal observational cohort study utilized data from the OAI cohort database, which includes a multicenter longitudinal cohort of 4,796 subjects with or at risk of OA (2004–2015 ClinicalTrials.gov identifier: NCT00080171) [15]. Participants were recruited from five clinical centers: The Ohio State University, University of Maryland, Johns Hopkins University, University of Pittsburgh, and Brown University/Memorial Hospital of Rhode Island. The study was compliant with the Health Insurance Portability and Accountability Act and received approval from the institutional review board at the University of California, San Francisco (approval number: 10-00532). All enrolled participants provided written informed consent.

**Fig. 1:**
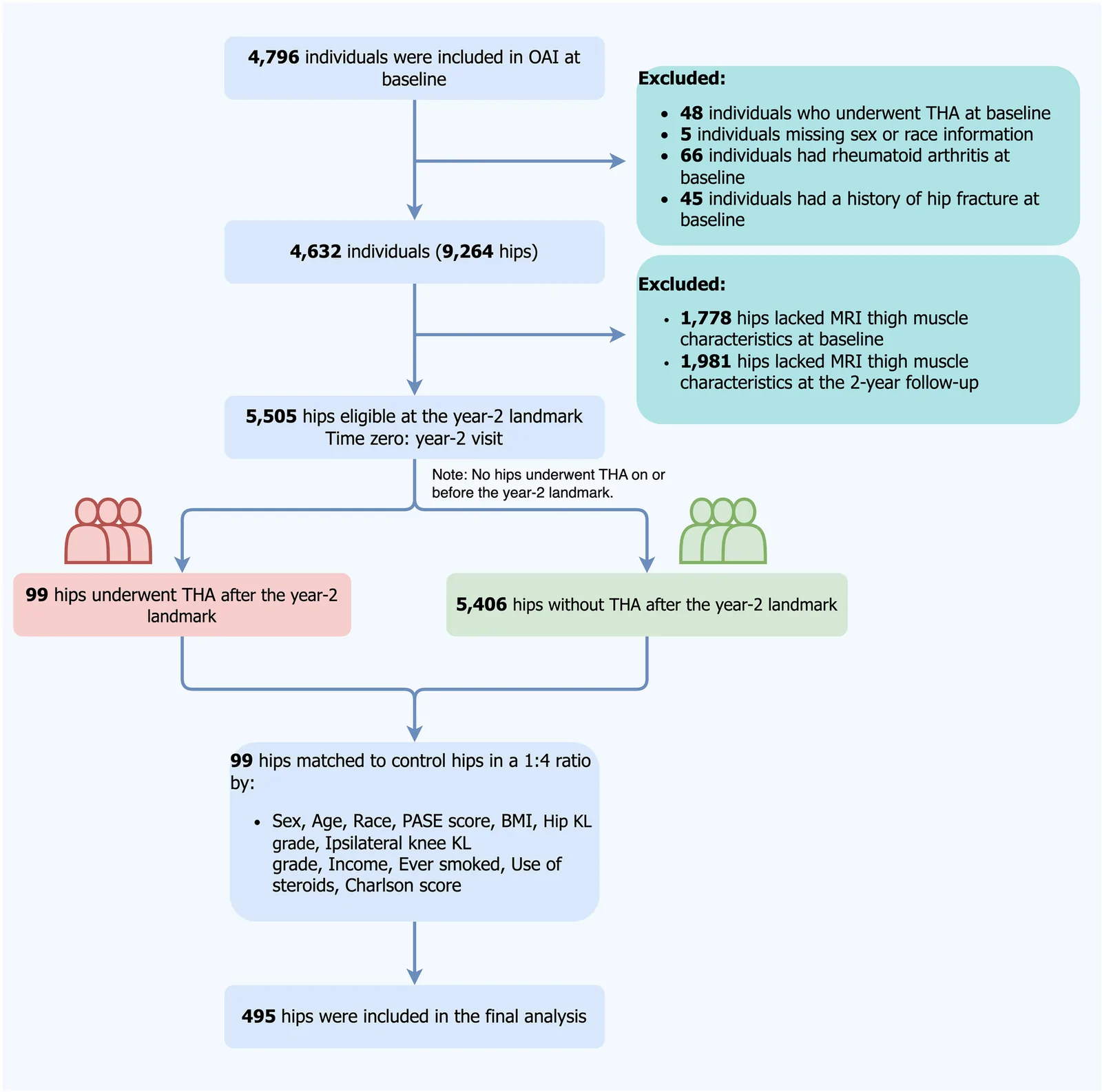
Flow diagram of hips included in the study. BMI = body mass index, PASE = Physical Activity Scale for the Elderly, THA = total hip arthroplasty, OAI = Osteoarthritis Initiative

As illustrated in Fig. 1, among the 4,796 participants enrolled in the OAI at baseline, individuals who had undergone THA at baseline, those with missing sex or race information, those with confirmed rheumatoid arthritis, and those with a history of hip fracture were excluded, leaving 4,632 participants (9,264 hips). Hips without both baseline and year-2 thigh MRI measurements were subsequently excluded. Because the primary exposure was the 2-year change in thigh MRI measures, we applied a year-2 landmark design and included only hips that remained free of THA at the year-2 visit. Among the 5,505 hips with both baseline and year-2 MRI measurements, none underwent THA on or before the year-2 landmark. During post-landmark follow-up through OAI year 9, 99 hips underwent THA. After 1:4 propensity score matching between THA cases and eligible controls, 495 hips were included in the final analysis.

### Propensity score matching and multiple imputation strategy

The extent of missing data was assessed in the eligible year-2 landmark cohort before propensity score matching (PSM) and multiple imputation. For each baseline covariate, the number and proportion of missing observations were calculated. We also reported the proportion of hips with at least one missing baseline covariate and the overall proportion of missing data fields across all baseline covariates.

Missing baseline covariates were imputed using multiple imputation by chained equations implemented with the “mice” package in R. Twenty imputed datasets were generated, with 50 iterations performed for each dataset. Predictive mean matching was used for continuous variables, including body mass index (BMI) and Physical Activity Scale for the Elderly (PASE) score. Binary logistic regression was used for binary categorical variables, whereas ordinal or multinomial logistic regression was used for categorical variables with more than two levels, as appropriate. The imputation model included all baseline covariates used for PSM, baseline and year-2 thigh MRI measures, 2-year changes in the MRI measures, THA event status, and an estimate of the cumulative baseline hazard. Participant ID and THA event status were included as predictor variables but were not imputed.

Within each imputed dataset, propensity scores were estimated using a multivariable logistic regression model including baseline age, sex, race, body mass index, Physical Activity Scale for the Elderly (PASE) score [16], hip and ipsilateral knee Kellgren-Lawrence (KL) grades [17], income, smoking history, steroid use, and Charlson comorbidity score [18]. THA case hips were matched to control hips at a 1:4 ratio using nearest-neighbor matching without replacement and without a caliper. Propensity score estimation, matching, and Cox proportional hazards analyses were performed separately within each imputed dataset. Covariate balance before and after matching was assessed using absolute standardized mean differences, with an absolute standardized mean difference below 0.10 indicating adequate balance, and was visualized using a Love plot. Cox models were stratified by propensity score-matched subclass, and robust sandwich standard errors clustered by participant ID were used to account for the matched design and within-participant correlation. The resulting log hazard ratios and standard errors were combined across imputed datasets using Rubin’s rules.

### Clinical outcomes

THA was used as the observed endpoint and as a marker of advanced hip disease. THA events were identified through centrally adjudicated replacements based on medical records forwarded by OAI clinical centers, identification on OAI study radiographs, or self-report. The analysis dataset did not contain sufficiently complete and consistent information on the surgical indication to reliably identify and exclude THA performed for osteonecrosis of the femoral head or other non-OA indications. Accordingly, all identified THA events meeting the prespecified case definition were included, and possible outcome misclassification was considered in the interpretation. The case group comprised hips that underwent THA after the year-2 landmark. The actual year-2 visit date was defined as time zero, and follow-up continued until ipsilateral THA or the last available OAI follow-up contact.

### Quantitative features of thigh MRI

**Fig. 2:**
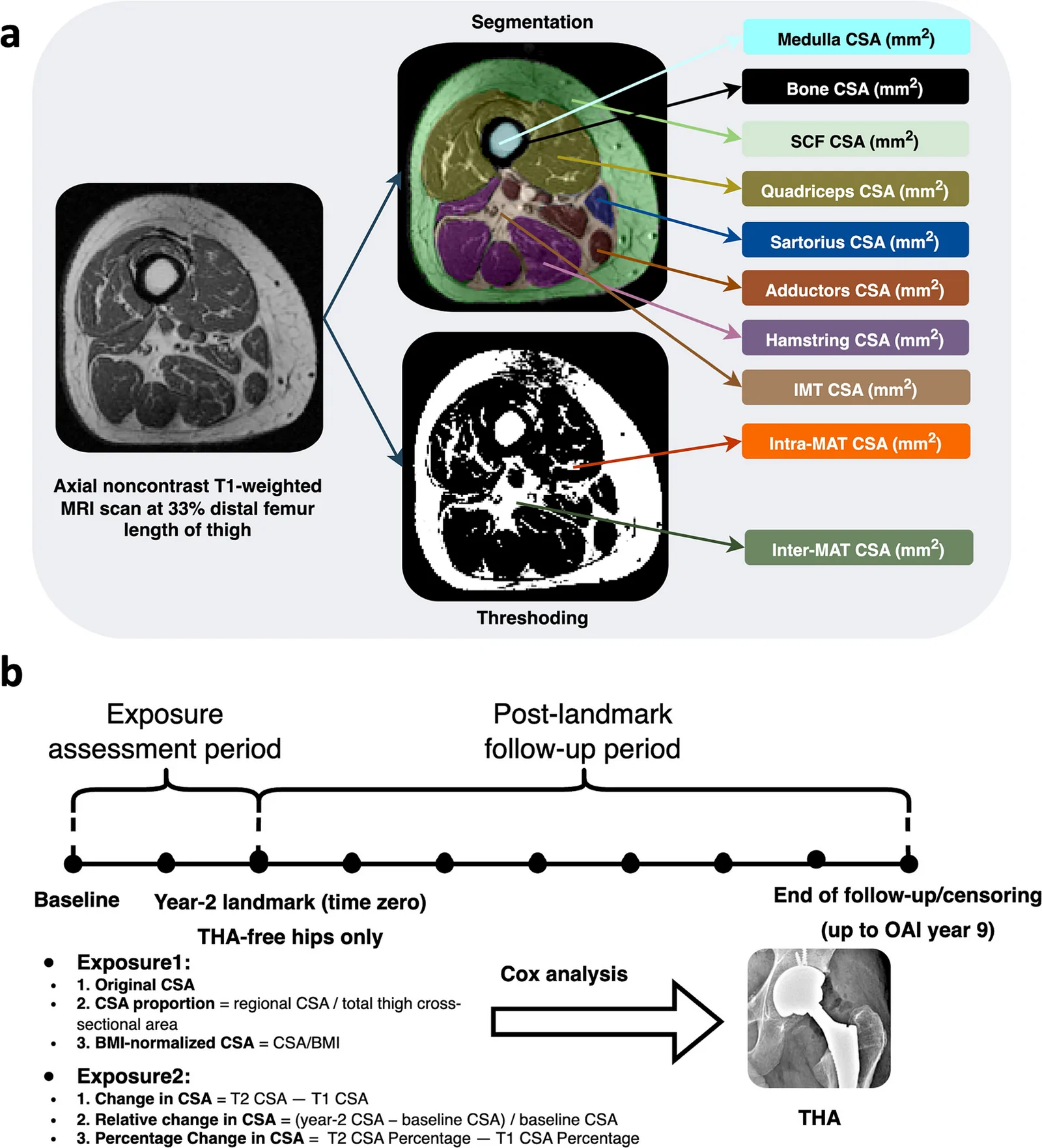
Study design diagram. **a** Regions corresponding to each component of the thigh MRI. **b** Year-2 landmark design and calculation methods for six thigh MRI CSA features. Percentage change in CSA was calculated as the year-2 CSA proportion minus the baseline CSA proportion and was expressed in percentage points rather than as a relative percent change. BMI = body mass index, CSA = cross-sectional area, THA = total hip arthroplasty

Based on Mohajer et al.’s secondary analysis of thigh muscle MRI in the OAI database [19], quantitative features of thigh muscle MRI at baseline and year 2 were measured on axial T1-weighted MRI images at 33% of the distal femur length. The imaging protocols for MRI can be found in Supplementary Methods. They trained and validated a U-Net network for thigh MRI segmentation and computed the CSA for subcutaneous fat and each thigh muscle group (quadriceps, hamstrings, adductors, and sartorius), as well as intramuscular adipose tissue (intra-MAT) for each muscle group and intermuscular adipose tissue (inter-MAT) [19]. Figure 2a illustrates the regions corresponding to each component in thigh MRI. Specific details of the model and algorithms are provided in their paper [19]. Features of thigh MRI in OAI patients are available at https://forms.gle/2U7dK6TvAN5uh8UW9, and we have obtained permission to reanalyze the data they provided.

Six thigh MRI feature-processing approaches were evaluated. Baseline measures included original CSA, CSA proportion (regional CSA divided by total thigh cross-sectional area), and BMI-normalized CSA (CSA divided by BMI) [19]. Longitudinal measures included the 2-year change in CSA (year-2 minus baseline CSA), relative change in CSA ([year-2 CSA − baseline CSA]/baseline CSA), and percentage change in CSA (year-2 CSA proportion minus baseline CSA proportion, expressed in percentage points rather than as a relative percent change) [20], as detailed in Fig. 2b. The 2-year change in total thigh muscle CSA was prespecified as the primary exposure because it represents overall longitudinal change in thigh muscle mass. All other muscle regions, tissue compartments, baseline measures, and processing approaches were treated as secondary or exploratory exposures.

### Sensitivity analyses

Three sensitivity analyses were conducted to assess the robustness of the findings. First, the analyses were repeated using complete cases after excluding hips with missing covariate data. Second, propensity score matching was repeated using a 1:3 case-to-control ratio. Third, to eliminate within-participant correlation between hips, one hip was randomly selected per participant before propensity score matching, after which the matching and Cox analyses were repeated. The direction, magnitude, and precision of the effect estimates from these sensitivity analyses were compared with those from the primary analysis.

### Statistical analysis

Baseline characteristics were summarized as means with standard deviations (SDs) for continuous variables and frequencies with percentages for categorical variables. Continuous and categorical variables were compared between the THA case and control groups using the Kruskal–Wallis test and chi-square test, respectively. Covariate balance after propensity score matching was primarily evaluated using absolute standardized mean differences (SMDs), with an absolute SMD below 0.10 considered indicative of adequate balance.

To facilitate comparisons across thigh MRI features with different units and scales, all MRI features were standardized using z scores [21]. Univariable Cox proportional hazards models were fitted separately for each standardized MRI feature. The actual year-2 visit date was defined as time zero, and follow-up time was calculated from the year-2 visit to ipsilateral THA or censoring at the last available follow-up. The Cox models were stratified by propensity score-matched subclass to account for the matched-set structure. Because some participants contributed both hips, robust sandwich standard errors clustered by participant ID were used to account for within-participant correlation.

Hazard ratios (HRs) with 95% confidence intervals (CIs) were reported per 1-SD increase in each MRI feature. An HR greater than 1 indicated a higher incidence of subsequent THA with increasing values of the MRI feature, whereas an HR below 1 indicated a lower incidence of subsequent THA. The proportional hazards assumption was assessed using Schoenfeld residuals.

In an exploratory analysis, the R package “survminer” was used to identify data-derived cutpoints for 2-year CSA changes in the quadriceps, hamstrings, adductors, and total muscle. Because cutpoint selection and evaluation were performed in the same dataset without internal or external validation, these estimates are reported only in Supplementary Table S7 as hypothesis-generating results and are not interpreted as clinically validated thresholds.

The primary exposure was evaluated using a two-sided *p*-value, with *p* < 0.05 considered statistically significant. To address multiple comparisons, the 95 secondary MRI tests were treated as one family, and their *p-values* were adjusted using the Benjamini–Hochberg FDR procedure. Both nominal *p*-values and FDR-adjusted *q*-values were reported for secondary analyses; *q* < 0.05 was considered statistically significant. Secondary findings with nominal *p* < 0.05 but *q* ≥ 0.05 were interpreted as exploratory. All statistical analyses were performed using R version 4.3.0, and all tests were two-sided.

## Results

### Sample characteristics

A total of 5,505 hips had both baseline and year-2 MRI measurements and an available year-2 visit date. None underwent THA on or before the year-2 landmark. During post-landmark follow-up, 99 hips met the THA case definition. After 1:4 propensity score matching, 495 hips, comprising 99 THA cases and 396 matched controls, were included in the final analysis. These hips were contributed by 450 participants; 405 participants (90.0%) contributed one hip and 45 (10.0%) contributed both hips. The distribution of THA events across post-landmark follow-up intervals is reported in Supplementary Table S2: 8, 11, 13, 15, 17, 19, and 16 events occurred during OAI years 2–3, 3–4, 4–5, 5–6, 6–7, 7–8, and 8–9, respectively.

As shown in Table 1, the mean age of the matched cohort was 63.9 years (SD, 8.7), and 61.2% of the included hips were contributed by female participants. The mean BMI was 28.1 kg/m^2^ (SD, 4.8), and the mean PASE score was 163.9 (SD, 83.5). Covariate balance improved substantially after propensity score matching. All baseline covariates had absolute standardized mean differences (SMDs) below 0.10 after matching, indicating adequate balance between THA cases and matched controls. Postmatching SMDs are presented in Table 1; SMDs before and after matching are visualized in the Love plot in Supplementary Fig. S1.

**Table 1:** Baseline characteristics of the study participants

	**Overall**	**Matched Cohort**			
**Characteristic**	**Overall** ***N* = 495 hips**	**Matched controls** ***N* = 396 hips**	**THA cases** ***N* = 99 hips**	***p*-value**	**Post-match SMD**
**Age, years**	63.9 (8.7)	64.0 (8.7)	63.7 (9.0)	0.82	0.034
**Race**				0.64	0.052
non-White	44 (8.9%)	34 (8.6%)	10 (10.1%)		
White	451 (91.1%)	362 (91.4%)	89 (89.9%)		
**Sex**				0.91	0.010
Female	303 (61.2%)	242 (61.1%)	61 (61.6%)		
Male	192 (38.8%)	154 (38.9%)	38 (38.4%)		
**Hip KL grade**				0.81	0.061
0	68 (13.7%)	54 (13.6%)	14 (14.1%)		
1–2	297 (60.0%)	240 (60.6%)	57 (57.6%)		
> 2	130 (26.3%)	102 (25.8%)	28 (28.3%)		
**Ipsilateral knee KL grade**				0.75	0.086
0	246 (49.7%)	194 (49.0%)	52 (52.5%)		
1–2	140 (28.3%)	112 (28.3%)	28 (28.3%)		
> 2	109 (22.0%)	90 (22.7%)	19 (19.2%)		
**BMI, kg/m**^**2**^	28.1 (4.8)	28.1 (4.9)	28.1 (4.4)	0.92	0.000
**PASE**	163.9 (83.5)	163.0 (81.8)	167.6 (90.3)	0.87	0.055
**Annual income**				0.96	0.004
< 50 K	146 (29.5%)	117 (29.5%)	29 (29.3%)		
≥ 50 K	349 (70.5%)	279 (70.5%)	70 (70.7%)		
**Ever smoked**	128 (25.9%)	103 (26.0%)	25 (25.3%)	0.90	0.016
**Use of steroids**	24 (4.8%)	19 (4.8%)	5 (5.1%)	0.93	0.014
**Charlson score**				0.91	0.054
0	390 (78.8%)	311 (78.5%)	79 (79.8%)		
1	86 (17.4%)	69 (17.4%)	17 (17.2%)		
> 1	19 (3.8%)	16 (4.0%)	3 (3.0%)		

BMI = body mass index; PASE = Physical Activity Scale for the Elderly; KL = Kellgren-Lawrence; SMD = standardized mean difference. Values are mean (SD) or n (%). An absolute SMD below 0.10 indicates adequate covariate balance after matching

### Missing data

Among the 5,505 hips eligible at the year-2 landmark, 991 (18.0%) had missing data for at least one baseline covariate included in the propensity score model. Across all baseline covariate data fields, the overall proportion of missing values was 2.4%. Income had the highest proportion of missing data (10.3%), followed by PASE score (6.8%) and ipsilateral knee KL grade (4.2%). The proportion of missing data was below 3% for each remaining covariate. The number and proportion of missing observations for each baseline covariate are reported in Supplementary Table S1.

### Quantitative thigh MRI features

As shown in Table 2, the mean total muscle CSA at baseline was 9,524.5 mm^2^ (SD, 2,580.5), mean total muscle IntraMAT was 430.9 mm^2^ (SD, 316.5), and mean InterMAT was 1,085.7 mm^2^ (SD, 394.3). Year-2 MRI values and 2-year changes are presented in Supplementary Table S3. Mean total muscle CSA was 9,325.0 mm^2^ (SD, 2,528.9) at year 2, corresponding to a mean 2-year change of − 199.5 mm^2^ (SD, 430.0). Mean total muscle IntraMAT increased to 462.1 mm^2^ (SD, 310.2), and mean InterMAT increased to 1,105.1 mm^2^ (SD, 386.4). The mean 2-year changes were − 99.0 mm^2^ for the quadriceps, − 64.0 mm^2^ for the hamstrings, and − 30.0 mm^2^ for the adductors.

**Table 2:** Quantitative thigh MRI features at baseline

MRI region, CSA (mm^2^)	Overall, *N* = 495 hips	Matched controls, *N* = 396 hips	THA cases, *N* = 99 hips	*p-*value
Medulla	241.0 (94.7)	243.1 (95.4)	232.4 (91.5)	0.32
Bone	422.6 (78.0)	422.1 (78.2)	424.9 (77.2)	0.81
SCF	7,089.9 (3,805.7)	7,135.7 (3,870.8)	6,906.7 (3,545.6)	0.83
Quadriceps	4,884.5 (1,334.0)	4,891.0 (1,317.9)	4,858.6 (1,403.2)	0.62
Hamstrings	3,191.7 (861.3)	3,185.2 (852.4)	3,217.5 (899.9)	0.92
Adductors	1,098.9 (587.4)	1,101.9 (582.9)	1,086.9 (608.3)	0.61
Sartorius	349.4 (125.5)	345.9 (122.6)	363.2 (136.4)	0.46
InterMAT	1,085.7 (394.3)	1,087.8 (394.7)	1,077.3 (394.5)	0.86
Quadriceps IntraMAT	164.2 (121.6)	166.5 (126.8)	155.0 (98.1)	0.74
Hamstrings IntraMAT	173.3 (162.4)	178.4 (173.0)	152.8 (109.0)	0.32
Adductors IntraMAT	64.5 (50.7)	65.6 (51.2)	60.2 (48.8)	0.35
Sartorius IntraMAT	29.0 (24.3)	29.2 (25.1)	28.1 (21.0)	0.81
IMT	1,474.8 (407.4)	1,474.6 (407.8)	1,475.3 (407.7)	0.92
Total fat	8,606.5 (4,014.1)	8,663.2 (4,083.0)	8,379.9 (3,736.9)	0.72
Total muscle IntraMAT	430.9 (316.5)	439.7 (331.9)	396.0 (243.5)	0.34
Total muscle	9,524.5 (2,580.5)	9,524.0 (2,554.9)	9,526.3 (2,694.2)	0.84

Values are mean (SD). CSA = cross-sectional area; InterMAT = intermuscular adipose tissue; IntraMAT = intramuscular adipose tissue; SCF = subcutaneous fat; SD = standard deviation

### Associations of baseline thigh MRI features with subsequent THA

**Fig. 3:**
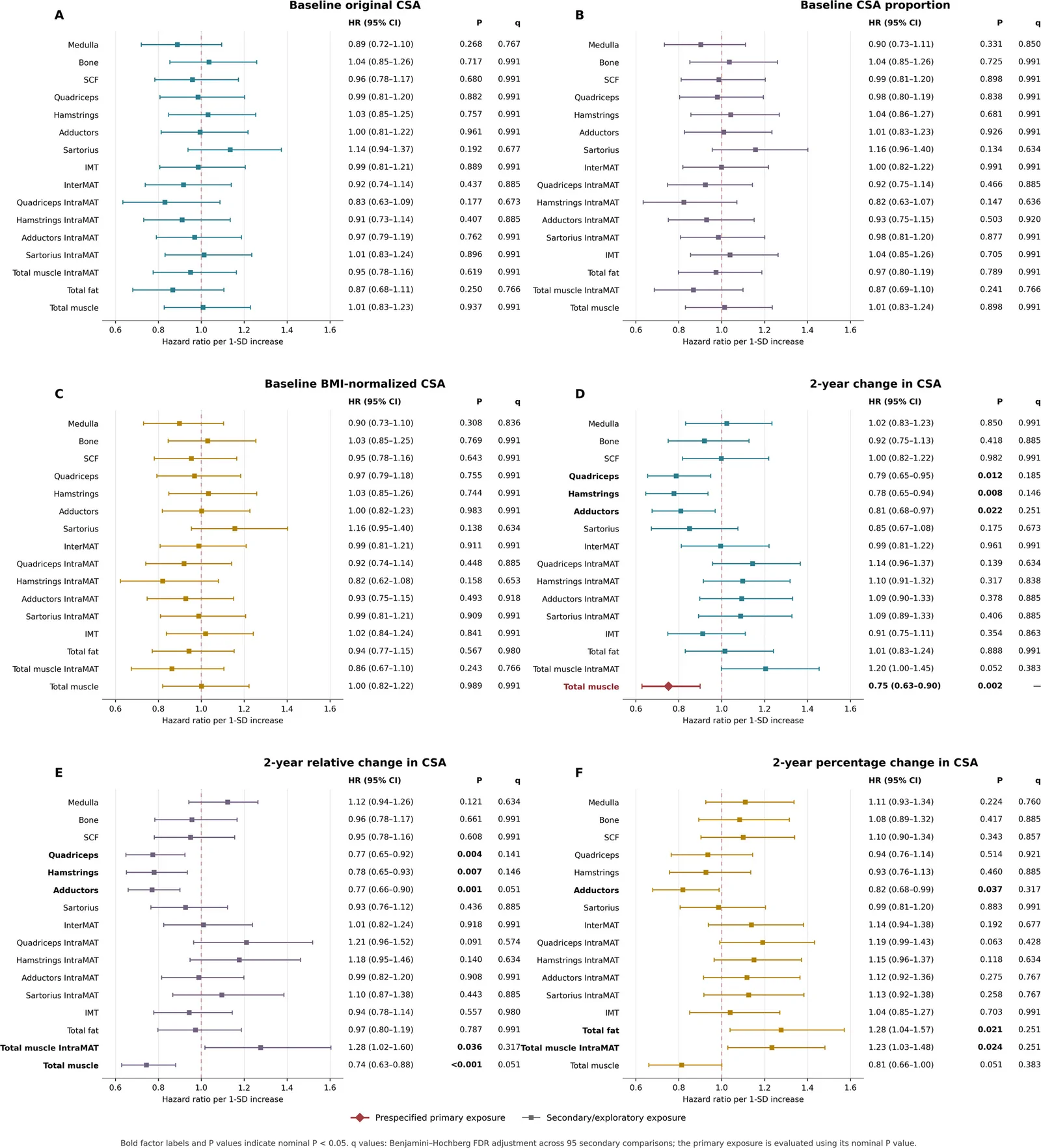
Associations of baseline and 2-year changes in thigh MRI measures with subsequent total hip arthroplasty. Hazard ratios (HRs) and 95% confidence intervals (CIs) are reported per 1-standard-deviation increase in each MRI measure. The prespecified primary exposure was the 2-year change in total thigh muscle CSA, indicated by a solid diamond in panel D. In panel F, percentage change in CSA represents the year-2 CSA proportion minus the baseline CSA proportion and is expressed in percentage points rather than as a relative percent change. Bold factor labels and *p*-values indicate nominal *p* < 0.05. All remaining associations were secondary or exploratory. Nominal *p*-values for the 95 secondary MRI comparisons were adjusted using the Benjamini–Hochberg false discovery rate procedure; *q* < 0.05 was considered statistically significant. CSA = cross-sectional area; CI = confidence interval; HR = hazard ratio; IMT = intermuscular tissue; InterMAT = intermuscular adipose tissue; IntraMAT = intramuscular adipose tissue; SCF = subcutaneous fat

In the secondary baseline analyses, none of the original CSA measures was associated with subsequent THA (Fig. 3A). Likewise, no association was identified when baseline CSA was expressed as a proportion or normalized by BMI (Fig. 3B and 3 C). These findings were unchanged after FDR correction.

### Associations of 2-year changes in thigh MRI features with subsequent THA

In the prespecified primary analysis, a greater 2-year change in total thigh muscle CSA was associated with a lower incidence of subsequent THA (HR per 1-SD increase, 0.75; 95% CI 0.63–0.90; *p* = 0.002; Fig. 3D). In secondary analyses, greater 2-year changes in quadriceps CSA (HR, 0.79; 95% CI 0.65–0.95; nominal *p* = 0.012; *q* = 0.185), hamstrings CSA (HR, 0.78; 95% CI 0.65–0.94; nominal *p* = 0.008; *q* = 0.146), and adductors CSA (HR, 0.81; 95% CI 0.68–0.97; nominal *p* = 0.022; *q* = 0.251) were nominally associated with a lower incidence of THA. None of these region-specific associations remained statistically significant after FDR correction. The 2-year changes in sartorius CSA, InterMAT, and the IntraMAT measures were also not significant after FDR correction.

The secondary analyses using relative change in CSA produced estimates in the same direction for total muscle and several individual muscle regions (Fig. 3E). The smallest adjusted *q*-values were observed for the relative changes in total muscle and adductors CSA (both *q* = 0.051); therefore, neither met the prespecified FDR significance threshold. For the percentage-change approach, nominal associations were observed for total fat and total muscle IntraMAT (nominal *p* = 0.021 and *p* = 0.024, respectively), but neither remained significant after FDR correction (both *q* = 0.251; Fig. 3F). Thus, no secondary MRI association was statistically significant after correction for the 95 secondary comparisons.

### Exploratory cutpoint analysis

Data-derived cutpoints for the 2-year CSA changes are reported in Supplementary Table S7. Because these cutpoints were selected and evaluated in the same dataset and were not internally or externally validated, they should be considered exploratory and should not be used for clinical decision-making.

### Sensitivity analysis results

The complete-case analysis and the analysis using 1:3 propensity score matching yielded results that were generally consistent with those of the primary analysis (Supplementary Tables S4 and S5). In the participant-clustered Cox models, the 2-year CSA changes in total muscle, quadriceps, hamstrings, and adductors were associated with subsequent THA, with HRs of 0.75 (95% CI 0.62–0.91; *p* = 0.004), 0.79 (95% CI 0.64–0.98; *p* = 0.031), 0.78 (95% CI 0.63–0.96; *p* = 0.020), and 0.81 (95% CI 0.66–0.99; *p* = 0.044), respectively.

In the sensitivity analysis restricted to one randomly selected hip per participant, the associations for total muscle CSA change (HR, 0.76; 95% CI 0.62–0.93; *p* = 0.007) and hamstrings CSA change (HR, 0.79; 95% CI 0.64–0.98; *p* = 0.032) remained statistically significant. The effect estimates for the quadriceps (HR, 0.80; 95% CI 0.64–1.00; *p* = 0.052) and adductors (HR, 0.83; 95% CI 0.67–1.03; *p* = 0.091) remained in the same direction but were no longer statistically significant. Detailed results are provided in Supplementary Table S6.

## Discussion

The relationship between longitudinal thigh muscle changes and hip OA progression remains poorly understood. In this year-2 landmark analysis of the OAI, the prespecified primary finding was that a greater 2-year change in total thigh muscle CSA—representing greater gain or less loss—was associated with a lower incidence of subsequent THA. Baseline MRI measures were not associated with THA. Although several individual muscle regions showed nominal associations, none of the 95 secondary comparisons remained statistically significant after FDR correction.

At baseline, thigh muscle CSA did not differ materially between the matched groups, and none of the baseline MRI measures was associated with subsequent THA. Results were similar when CSA was expressed as a proportion or normalized by BMI. A single baseline measurement may be affected by substantial between-person variation and may therefore provide limited information for risk stratification. The present study did not assess discrimination, calibration, time-dependent area under the curve, or internally validated prediction performance; consequently, the findings should not be interpreted as evidence of predictive superiority.

Regarding longitudinal CSA, the primary result suggests that overall thigh muscle change may contain prognostic information not captured by a single baseline measurement. The nominal inverse associations observed for the quadriceps, hamstrings, and adductors were directionally consistent with the primary result but did not remain significant after FDR correction and should therefore be considered exploratory. To our knowledge, few studies have examined longitudinal thigh muscle CSA in relation to hip OA outcomes. Previous studies have linked decreased quadriceps CSA and increased IntraMAT to knee OA progression [4, 5, 22]. Biologically, the thigh muscle groups contribute to lower-limb power, stability, and balance [23, 24], and muscle loss may co-occur with pain, reduced activity, joint instability, and further functional decline [25, 26]. Nevertheless, the present observational analysis cannot determine whether muscle loss causally increases THA risk or instead reflects worsening hip disease.

Nominal associations were observed for selected adipose-tissue measures under some processing approaches, but none remained significant after false discovery rate correction. These results do not provide conclusive evidence that InterMAT or IntraMAT is independently associated with subsequent THA. Such measures may capture aspects of muscle quality because muscle fat infiltration has been associated with metabolic dysfunction and chronic inflammation [27, 28], but their value for risk stratification in hip OA requires confirmation in independent cohorts.

The OAI is well suited to evaluate longitudinal muscle markers because of its large cohort, detailed covariate data, and extended follow-up. The data-derived cutpoints may be useful for hypothesis generation but are confined to the Supplementary Material and should not be considered clinically validated thresholds. Automated MRI segmentation could facilitate reproducible quantification of thigh muscle CSA in future studies. However, this observational study was not designed to determine whether muscle-strengthening exercise prevents hip OA progression or THA. Any potential benefit of exercise remains a hypothesis that requires testing in appropriately designed interventional trials.

This study has several limitations. First, although propensity score matching addressed measured covariates, residual and unmeasured confounding remains possible. Pain and functional decline may reduce physical activity and contribute to muscle loss; thus, reverse causation cannot be excluded. Second, MRI measurements were obtained from a single axial slice at the distal-thigh level and did not provide volumetric assessment. The OAI thigh protocol also did not include key hip-related muscles such as the gluteal musculature, limiting mechanistic inference regarding hip stability and loading. Third, the surgical indication was not consistently available, so THA for osteonecrosis of the femoral head or other non-OA indications could not be reliably identified and excluded. THA may also reflect disease severity, symptoms, patient preference, access to care, insurance, and overall health; therefore, it is an imperfect surrogate for hip OA progression. Fourth, the cutpoints were derived in the same sample without validation and may be optimistic. Fifth, the study did not evaluate prediction-model discrimination, calibration, or clinical utility. Finally, thigh muscle change may vary according to disease stage and time to surgery, and the present sample size limited precision for secondary analyses.

## Conclusion

In this OAI year-2 landmark analysis, a greater 2-year change in total thigh muscle CSA, corresponding to greater gain or less loss, was associated with a lower incidence of subsequent THA. Conversely, lower CSA change values or greater muscle loss over 2 years were associated with higher subsequent THA incidence. Associations involving individual muscle regions and alternative processing approaches did not remain significant after false discovery rate correction and should be interpreted as exploratory. Longitudinal total thigh muscle CSA may be a potential risk-stratification marker, but independent validation and formal assessment of predictive performance are required before clinical application.

## Supplementary Information


Supplementary Material 1.

## Data Availability

The datasets supporting the conclusions of this article are available from the Osteoarthritis Initiative (OAI) repository (https://nda.nih.gov/oai/) in accordance with the OAI data-use policies. The present analysis used de-identified data obtained from this repository.
