# Supplementary material for: Association of thigh muscle status and longitudinal changes assessed by MRI with subsequent total hip arthroplasty: insights from the osteoarthritis initiative
Source: Arthroplasty. 2026 Sep 1;8:64. doi: 10.1186/s42836-026-00423-5 (PMC13531860; doi:10.1186/s42836-026-00423-5)
Supplement: Supplementary file 1 [file 42836_2026_423_MOESM1_ESM.docx]

**Supplementary material**

**Part1**

**Methods: Thigh MRI Acquisition Protocol**

The protocol was optimized for segmentation of subcutaneous and intermuscular fat depots, skeletal muscle, and specific muscle groups. Thigh MRI consisted of a 15-section contiguous axial T1-weighted acquisition of the quadriceps region centered 100 mm above the medial femoral epiphysis (section thickness, 5 mm; in-plane resolution, 0.98 mm or 0.74 mm; echo time, 10 ms; repetition time, 500 ms; OAI image datasets 0.E.1, 3.E.1, and 6.E.1).

**Part 2**

**Table S1.** Missingness of baseline covariates before multiple imputation

| **Baseline covariate** | **Missing, *n*** | **Missing, %** |
| --- | --- | --- |
| Age | 0 | 0.0 |
| Sex | 0 | 0.0 |
| Race | 0 | 0.0 |
| BMI | 83 | 1.5 |
| PASE score | 374 | 6.8 |
| Hip KL grade | 0 | 0.0 |
| Ipsilateral knee KL grade | 231 | 4.2 |
| Income | 566 | 10.3 |
| Ever smoked | 88 | 1.6 |
| Steroid use | 132 | 2.4 |
| Charlson score | 0 | 0.0 |

Missingness was evaluated among 5,505 hips eligible at the year-2 landmark before propensity score matching and multiple imputation. Overall, 991 hips (18.0%) had missing data for at least one baseline covariate. Of the 60,555 covariate data fields evaluated, 1,474 (2.4%) were missing. BMI, body mass index; KL, Kellgren–Lawrence; PASE, Physical Activity Scale for the Elderly.

**Table S2.** Distribution of THA events after the year-2 landmark

| **OAI follow-up interval** | **THA events, n** | **THA events, %** | **Cumulative events, *n*** |
| --- | --- | --- | --- |
| Year 2 to <3 | 8 | 8.1 | 8 |
| Year 3 to <4 | 11 | 11.1 | 19 |
| Year 4 to <5 | 13 | 13.1 | 32 |
| Year 5 to <6 | 15 | 15.2 | 47 |
| Year 6 to <7 | 17 | 17.2 | 64 |
| Year 7 to <8 | 19 | 19.2 | 83 |
| Year 8 to 9 | 16 | 16.2 | 99 |

Interval-specific event counts sum to 99. THA = total hip arthroplasty.

**Table S3.** Quantitative thigh MRI features at year 2 and their 2-year changes

**A. Year-2 MRI values**

| **MRI region, CSA (mm²)** | **Overall, *N* = 495 hips** | **Matched controls, *N* = 396 hips** | **THA cases, *N* = 99 hips** | ***p*-value** |
| --- | --- | --- | --- | --- |
| Medulla | 238.2 (92.8) | 241.1 (93.5) | 226.4 (90.6) | 0.12 |
| Bone | 420.8 (76.4) | 421.1 (76.6) | 419.9 (76.4) | 0.09 |
| SCF | 7,144.9 (3,729.6) | 7,195.7 (3,793.4) | 6,941.7 (3,510.1) | 0.48 |
| Quadriceps | 4,785.5 (1,307.3) | 4,811.0 (1,291.5) | 4,683.6 (1,389.2) | 0.018 |
| Hamstrings | 3,127.7 (844.1) | 3,135.2 (835.4) | 3,097.5 (890.9) | 0.011 |
| Adductors | 1,068.9 (575.7) | 1,079.9 (571.2) | 1,026.9 (602.2) | 0.026 |
| Sartorius | 342.8 (123.0) | 340.9 (120.1) | 350.2 (135.0) | 0.14 |
| InterMAT | 1,105.1 (386.4) | 1,105.8 (386.8) | 1,102.3 (390.6) | 0.48 |
| Quadriceps IntraMAT | 175.8 (119.2) | 176.5 (124.3) | 173.0 (97.1) | 0.09 |
| Hamstrings IntraMAT | 186.1 (159.2) | 189.4 (169.5) | 172.8 (107.9) | 0.11 |
| Adductors IntraMAT | 69.1 (49.7) | 69.6 (50.2) | 67.2 (48.3) | 0.16 |
| Sartorius IntraMAT | 30.7 (23.8) | 30.7 (24.6) | 30.6 (20.8) | 0.31 |
| IMT | 1,510.8 (399.3) | 1,506.6 (399.6) | 1,527.3 (403.6) | 0.17 |
| Total fat | 8,709.1 (3,933.8) | 8,755.2 (4,001.3) | 8,524.9 (3,699.5) | 0.21 |
| Total muscle IntraMAT | 462.1 (310.2) | 466.7 (325.3) | 444.0 (241.1) | 0.08 |
| Total muscle | 9,325.0 (2,528.9) | 9,367.0 (2,503.8) | 9,158.3 (2,667.3) | 0.004 |

**B. Two-year changes in MRI values**

| **MRI region, CSA (mm²)** | **Overall, *N* = 495 hips** | **Matched controls, *N* = 396 hips** | **THA cases, *N* = 99 hips** | ***p*-value** |
| --- | --- | --- | --- | --- |
| Medulla | −2.8 (19.0) | −2.0 (18.0) | −6.0 (22.0) | 0.12 |
| Bone | −1.8 (15.5) | −1.0 (15.0) | −5.0 (17.0) | 0.09 |
| SCF | 55.0 (618.0) | 60.0 (610.0) | 35.0 (650.0) | 0.48 |
| Quadriceps | −99.0 (324.0) | −80.0 (310.0) | −175.0 (360.0) | 0.018 |
| Hamstrings | −64.0 (231.0) | −50.0 (220.0) | −120.0 (255.0) | 0.011 |
| Adductors | −30.0 (158.0) | −22.0 (150.0) | −60.0 (180.0) | 0.026 |
| Sartorius | −6.6 (39.5) | −5.0 (38.0) | −13.0 (44.0) | 0.14 |
| InterMAT | 19.4 (123.0) | 18.0 (120.0) | 25.0 (135.0) | 0.48 |
| Quadriceps IntraMAT | 11.6 (44.0) | 10.0 (42.0) | 18.0 (50.0) | 0.09 |
| Hamstrings IntraMAT | 12.8 (52.0) | 11.0 (50.0) | 20.0 (57.0) | 0.11 |
| Adductors IntraMAT | 4.6 (18.8) | 4.0 (18.0) | 7.0 (21.0) | 0.16 |
| Sartorius IntraMAT | 1.7 (8.2) | 1.5 (8.0) | 2.5 (9.0) | 0.31 |
| IMT | 36.0 (130.0) | 32.0 (125.0) | 52.0 (145.0) | 0.17 |
| Total fat | 102.6 (655.0) | 92.0 (640.0) | 145.0 (710.0) | 0.21 |
| Total muscle IntraMAT | 31.2 (112.0) | 27.0 (105.0) | 48.0 (135.0) | 0.08 |
| Total muscle | −199.5 (430.0) | −157.0 (405.0) | −368.0 (490.0) | 0.004 |

Values are mean (SD). CSA = cross-sectional area; IMT = intermuscular tissue; InterMAT = intermuscular adipose tissue; IntraMAT = intramuscular adipose tissue; SCF = subcutaneous fat; SD = standard deviation.

**Table S4.** Complete-Case Risk Analysis after exclusion of hips with missing covariate data

| **Risk Analysis of Quantitative Thigh MRI Features at Baseline** | | | | **Risk Analysis of Quantitative Changes in** **Thigh MRI Features Over 2 Years** | | | |
| --- | --- | --- | --- | --- | --- | --- | --- |
| **Original CSA** | **MRI region** | **HR (95% CI)** | **P** | **2-year change in CSA** | **MRI region** | **HR (95% CI)** | **P** |
|  | Medulla | 0.92 (0.75, 1.14) | 0.46 |  | Medulla | 1.15 (0.97, 1.36) | 0.1 |
|  | Bone | 1.13 (0.93, 1.36) | 0.22 |  | Bone | 0.83 (0.68, 1.02) | 0.078 |
|  | SCF | 1.02 (0.84, 1.24) | 0.85 |  | SCF | 0.99 (0.81, 1.22) | 0.94 |
|  | Quadriceps | 1.09 (0.90, 1.32) | 0.39 |  | Quadriceps | 0.81 (0.68, 0.97) | **0.026** |
|  | Hamstrings | 1.11 (0.92, 1.34) | 0.27 |  | Hamstrings | 0.78 (0.64, 0.95) | **0.011** |
|  | Adductors | 1.00 (0.82, 1.22) | 0.98 |  | Adductors | 0.80 (0.66, 0.97) | **0.022** |
|  | Sartorius | 1.03 (0.85, 1.26) | 0.76 |  | Sartorius | 0.93 (0.77, 1.12) | 0.44 |
|  | InterMAT | 1.03 (0.85, 1.26) | 0.74 |  | InterMAT | 1.06 (0.86, 1.31) | 0.59 |
|  | QuadIntraMAT | 0.99 (0.81, 1.21) | 0.95 |  | QuadIntraMAT | 1.21 (1.01, 1.46) | **0.043** |
|  | HamstringIntraMAT | 0.97 (0.79, 1.19) | 0.78 |  | HamstringIntraMAT | 1.05 (0.87, 1.27) | 0.62 |
|  | AdductorIntraMAT | 1.02 (0.84, 1.25) | 0.81 |  | AdductorIntraMAT | 1.07 (0.88, 1.31) | 0.49 |
|  | SartoriusIntraMAT | 1.05 (0.87, 1.26) | 0.62 |  | SartoriusIntraMAT | 1.08 (0.89, 1.33) | 0.44 |
|  | IMT | 1.08 (0.89, 1.30) | 0.46 |  | IMT | 0.92 (0.75, 1.12) | 0.41 |
|  | Total fat | 1.02 (0.84, 1.24) | 0.84 |  | Total fat | 1.02 (0.83, 1.25) | 0.87 |
|  | Total muscle IntraMAT | 0.99 (0.81, 1.21) | 0.94 |  | Total muscle IntraMAT | 1.18 (0.97, 1.43) | 0.091 |
|  | Total muscle | 1.09 (0.90, 1.32) | 0.37 |  | Total muscle | 0.77 (0.65, 0.92) | **0.004** |
| **CSA proportion** | Medulla | 0.90 (0.72, 1.11) | 0.31 | **Relative change in CSA over 2 years** | Medulla | 1.21 (0.97, 1.51) | 0.089 |
|  | Bone | 1.02 (0.83, 1.24) | 0.87 |  | Bone | 0.86 (0.70, 1.05) | 0.14 |
|  | SCF | 0.98 (0.80, 1.19) | 0.8 |  | SCF | 0.98 (0.80, 1.20) | 0.82 |
|  | Quadriceps | 1.02 (0.83, 1.24) | 0.86 |  | Quadriceps | 0.82 (0.69, 0.99) | **0.036** |
|  | Hamstrings | 1.05 (0.86, 1.28) | 0.62 |  | Hamstrings | 0.79 (0.65, 0.95) | **0.015** |
|  | Adductors | 0.98 (0.80, 1.19) | 0.81 |  | Adductors | 0.80 (0.67, 0.94) | **0.006** |
|  | Sartorius | 1.03 (0.85, 1.26) | 0.76 |  | Sartorius | 0.96 (0.80, 1.16) | 0.68 |
|  | InterMAT | 0.97 (0.79, 1.18) | 0.75 |  | InterMAT | 1.05 (0.85, 1.30) | 0.63 |
|  | QuadIntraMAT | 0.96 (0.79, 1.18) | 0.72 |  | QuadIntraMAT | 1.25 (0.99, 1.59) | 0.064 |
|  | HamstringIntraMAT | 0.93 (0.75, 1.15) | 0.49 |  | HamstringIntraMAT | 1.04 (0.85, 1.27) | 0.69 |
|  | AdductorIntraMAT | 1.02 (0.84, 1.25) | 0.83 |  | AdductorIntraMAT | 1.01 (0.83, 1.23) | 0.93 |
|  | SartoriusIntraMAT | 1.04 (0.86, 1.26) | 0.69 |  | SartoriusIntraMAT | 1.13 (0.89, 1.44) | 0.32 |
|  | IMT | 1.00 (0.82, 1.22) | 0.98 |  | IMT | 0.95 (0.78, 1.16) | 0.59 |
|  | Total fat | 0.97 (0.79, 1.18) | 0.74 |  | Total fat | 1.00 (0.81, 1.23) | 1 |
|  | Total muscle IntraMAT | 0.95 (0.78, 1.17) | 0.66 |  | Total muscle IntraMAT | 1.21 (0.97, 1.50) | 0.085 |
|  | Total muscle | 1.03 (0.85, 1.26) | 0.75 |  | Total muscle | 0.77 (0.64, 0.91) | **0.003** |
| **BMI-normalized CSA** | Medulla | 0.91 (0.74, 1.12) | 0.38 | **Percentage change in CSA over 2 years** | Medulla | 1.18 (0.99, 1.40) | 0.13 |
|  | Bone | 1.06 (0.87, 1.29) | 0.54 |  | Bone | 1.00 (0.82, 1.22) | 0.071 |
|  | SCF | 0.98 (0.80, 1.19) | 0.82 |  | SCF | 1.14 (0.93, 1.41) | 0.064 |
|  | Quadriceps | 1.06 (0.87, 1.29) | 0.57 |  | Quadriceps | 0.93 (0.75, 1.14) | 0.81 |
|  | Hamstrings | 1.10 (0.90, 1.34) | 0.34 |  | Hamstrings | 0.91 (0.74, 1.12) | 0.21 |
|  | Adductors | 1.00 (0.82, 1.22) | 0.99 |  | Adductors | 0.80 (0.66, 0.98) | 0.48 |
|  | Sartorius | 1.03 (0.85, 1.26) | 0.66 |  | Sartorius | 1.07 (0.87, 1.32) | 0.39 |
|  | InterMAT | 1.01 (0.83, 1.23) | 0.92 |  | InterMAT | 1.18 (0.97, 1.45) | **0.027** |
|  | QuadIntraMAT | 0.98 (0.80, 1.20) | 0.87 |  | QuadIntraMAT | 1.19 (0.99, 1.43) | 0.52 |
|  | HamstringIntraMAT | 0.95 (0.77, 1.17) | 0.64 |  | HamstringIntraMAT | 1.08 (0.89, 1.31) | 0.1 |
|  | AdductorIntraMAT | 1.03 (0.84, 1.25) | 0.78 |  | AdductorIntraMAT | 1.09 (0.90, 1.33) | 0.063 |
|  | SartoriusIntraMAT | 1.06 (0.87, 1.27) | 0.57 |  | SartoriusIntraMAT | 1.10 (0.90, 1.36) | 0.42 |
|  | IMT | 1.06 (0.87, 1.28) | 0.58 |  | IMT | 1.03 (0.84, 1.26) | 0.38 |
|  | Total fat | 0.98 (0.80, 1.19) | 0.81 |  | Total fat | 1.22 (0.99, 1.50) | 0.34 |
|  | Total muscle IntraMAT | 0.98 (0.80, 1.20) | 0.84 |  | Total muscle IntraMAT | 1.19 (0.99, 1.43) | 0.78 |
|  | Total muscle | 1.07 (0.88, 1.31) | 0.47 |  | Total muscle | 0.85 (0.69, 1.05) | 0.063 |

CI = confidence interval; CSA = cross-sectional area; HR = hazard ratio; IMT = intermuscular tissue; InterMAT = intermuscular adipose tissue; IntraMAT = intramuscular adipose tissue; SCF = subcutaneous fat.

**Table S5. Risk Analysis using 1:3 propensity score matching**

| **Risk Analysis of Quantitative Thigh MRI Features at Baseline** | | | | **Risk Analysis of Quantitative Changes in** **Thigh MRI Features Over 2 Years** | | | |
| --- | --- | --- | --- | --- | --- | --- | --- |
| **Original CSA** | **MRI region** | **HR (95% CI)** | **P** | **2-year change in CSA** | **MRI region** | **HR (95% CI)** | **P** |
|  | Medulla | 0.90 (0.73, 1.11) | 0.31 |  | Medulla | 1.09 (0.95, 1.24) | 0.21 |
|  | Bone | 1.08 (0.90, 1.31) | 0.41 |  | Bone | 0.95 (0.79, 1.15) | 0.6 |
|  | SCF | 1.04 (0.85, 1.26) | 0.72 |  | SCF | 0.96 (0.78, 1.18) | 0.7 |
|  | Quadriceps | 1.02 (0.84, 1.25) | 0.81 |  | Quadriceps | 0.81 (0.69, 0.96) | **0.012** |
|  | Hamstrings | 1.05 (0.86, 1.27) | 0.65 |  | Hamstrings | 0.74 (0.62, 0.88) | **<0.001** |
|  | Adductors | 1.03 (0.85, 1.26) | 0.76 |  | Adductors | 0.80 (0.67, 0.94) | **0.007** |
|  | Sartorius | 1.09 (0.90, 1.31) | 0.39 |  | Sartorius | 0.86 (0.70, 1.05) | 0.14 |
|  | InterMAT | 0.94 (0.77, 1.16) | 0.57 |  | InterMAT | 0.97 (0.79, 1.19) | 0.76 |
|  | QuadIntraMAT | 0.80 (0.62, 1.03) | 0.08 |  | QuadIntraMAT | 1.06 (0.89, 1.27) | 0.5 |
|  | HamstringIntraMAT | 0.87 (0.68, 1.10) | 0.23 |  | HamstringIntraMAT | 1.05 (0.86, 1.27) | 0.64 |
|  | AdductorIntraMAT | 0.91 (0.73, 1.13) | 0.38 |  | AdductorIntraMAT | 0.99 (0.81, 1.20) | 0.91 |
|  | SartoriusIntraMAT | 0.97 (0.79, 1.20) | 0.8 |  | SartoriusIntraMAT | 1.03 (0.84, 1.27) | 0.74 |
|  | IMT | 0.99 (0.82, 1.21) | 0.96 |  | IMT | 0.88 (0.73, 1.05) | 0.16 |
|  | Total fat | 1.02 (0.83, 1.24) | 0.88 |  | Total fat | 0.97 (0.79, 1.19) | 0.76 |
|  | Total muscle IntraMAT | 0.84 (0.66, 1.06) | 0.14 |  | Total muscle IntraMAT | 1.07 (0.89, 1.28) | 0.5 |
|  | Total muscle | 1.04 (0.86, 1.26) | 0.7 |  | Total muscle | 0.78 (0.67, 0.90) | **<0.001** |
| **CSA proportion** | Medulla | 0.87 (0.71, 1.08) | 0.22 | **Relative change in CSA over 2 years** | Medulla | 1.18 (0.97, 1.43) | 0.091 |
|  | Bone | 1.00 (0.82, 1.22) | 0.99 |  | Bone | 1.02 (0.83, 1.24) | 0.87 |
|  | SCF | 1.02 (0.84, 1.24) | 0.85 |  | SCF | 0.90 (0.73, 1.10) | 0.28 |
|  | Quadriceps | 0.97 (0.79, 1.18) | 0.75 |  | Quadriceps | 0.83 (0.73, 0.95) | **0.007** |
|  | Hamstrings | 1.01 (0.83, 1.23) | 0.93 |  | Hamstrings | 0.77 (0.66, 0.91) | **0.002** |
|  | Adductors | 1.03 (0.85, 1.26) | 0.76 |  | Adductors | 0.92 (0.83, 1.03) | 0.14 |
|  | Sartorius | 1.07 (0.88, 1.30) | 0.48 |  | Sartorius | 0.88 (0.73, 1.07) | 0.19 |
|  | InterMAT | 0.88 (0.71, 1.08) | 0.21 |  | InterMAT | 0.96 (0.78, 1.17) | 0.66 |
|  | QuadIntraMAT | 0.79 (0.61, 1.01) | 0.063 |  | QuadIntraMAT | 1.23 (0.95, 1.58) | 0.11 |
|  | HamstringIntraMAT | 0.84 (0.66, 1.07) | 0.15 |  | HamstringIntraMAT | 1.09 (0.88, 1.33) | 0.43 |
|  | AdductorIntraMAT | 0.92 (0.74, 1.14) | 0.44 |  | AdductorIntraMAT | 1.03 (0.82, 1.28) | 0.82 |
|  | SartoriusIntraMAT | 0.98 (0.80, 1.20) | 0.84 |  | SartoriusIntraMAT | 1.10 (0.81, 1.50) | 0.53 |
|  | IMT | 0.93 (0.76, 1.13) | 0.46 |  | IMT | 0.87 (0.72, 1.04) | 0.12 |
|  | Total fat | 0.98 (0.81, 1.20) | 0.87 |  | Total fat | 0.90 (0.74, 1.10) | 0.32 |
|  | Total muscle IntraMAT | 0.81 (0.63, 1.04) | 0.096 |  | Total muscle IntraMAT | 1.16 (0.94, 1.43) | 0.18 |
|  | Total muscle | 1.00 (0.82, 1.22) | 0.97 |  | Total muscle | 0.83 (0.74, 0.92) | **<0.001** |
| **BMI-normalized CSA** | Medulla | 0.91 (0.74, 1.12) | 0.38 | **Percentage change in CSA over 2 years** | Medulla | 1.10 (0.97, 1.24) | 0.13 |
|  | Bone | 1.10 (0.91, 1.34) | 0.32 |  | Bone | 1.20 (0.98, 1.47) | 0.071 |
|  | SCF | 1.04 (0.85, 1.26) | 0.72 |  | SCF | 1.09 (0.90, 1.32) | 0.36 |
|  | Quadriceps | 1.04 (0.86, 1.27) | 0.68 |  | Quadriceps | 0.95 (0.78, 1.16) | 0.65 |
|  | Hamstrings | 1.09 (0.90, 1.33) | 0.38 |  | Hamstrings | 0.95 (0.78, 1.16) | 0.62 |
|  | Adductors | 1.07 (0.88, 1.31) | 0.5 |  | Adductors | 0.84 (0.72, 0.99) | **0.039** |
|  | Sartorius | 1.13 (0.94, 1.36) | 0.19 |  | Sartorius | 1.01 (0.83, 1.23) | 0.91 |
|  | InterMAT | 0.94 (0.76, 1.15) | 0.53 |  | InterMAT | 1.07 (0.91, 1.26) | 0.39 |
|  | QuadIntraMAT | 0.80 (0.63, 1.02) | 0.075 |  | QuadIntraMAT | 1.07 (0.90, 1.27) | 0.45 |
|  | HamstringIntraMAT | 0.86 (0.68, 1.09) | 0.22 |  | HamstringIntraMAT | 1.09 (0.90, 1.32) | 0.38 |
|  | AdductorIntraMAT | 0.93 (0.75, 1.16) | 0.53 |  | AdductorIntraMAT | 1.04 (0.85, 1.28) | 0.71 |
|  | SartoriusIntraMAT | 1.00 (0.82, 1.22) | 0.98 |  | SartoriusIntraMAT | 1.05 (0.86, 1.28) | 0.65 |
|  | IMT | 1.01 (0.83, 1.24) | 0.89 |  | IMT | 0.99 (0.81, 1.21) | 0.93 |
|  | Total fat | 1.02 (0.84, 1.24) | 0.86 |  | Total fat | 1.13 (0.94, 1.37) | 0.21 |
|  | Total muscle IntraMAT | 0.84 (0.66, 1.06) | 0.14 |  | Total muscle IntraMAT | 1.11 (0.93, 1.33) | 0.26 |
|  | Total muscle | 1.08 (0.88, 1.31) | 0.47 |  | Total muscle | 0.89 (0.74, 1.08) | 0.23 |

CI = confidence interval; CSA = cross-sectional area; HR = hazard ratio; IMT = intermuscular tissue; InterMAT = intermuscular adipose tissue; IntraMAT = intramuscular adipose tissue; SCF = subcutaneous fat.

**Table S6.** Analyses Accounting for participants contributing two hips

| **2-year MRI change** | **Participant-clustered Cox model, HR (95% CI)** | ***p*-value** | **One-hip-per-participant analysis, HR (95% CI)** | ***p*-value** |
| --- | --- | --- | --- | --- |
| Total muscle CSA | 0.75 (0.62–0.91) | 0.004 | 0.76 (0.62–0.93) | 0.007 |
| Quadriceps CSA | 0.79 (0.64–0.98) | 0.031 | 0.80 (0.64–1.00) | 0.052 |
| Hamstrings CSA | 0.78 (0.63–0.96) | 0.020 | 0.79 (0.64–0.98) | 0.032 |
| Adductors CSA | 0.81 (0.66–0.99) | 0.044 | 0.83 (0.67–1.03) | 0.091 |

HRs are reported per 1-standard-deviation increase in the 2-year CSA change. The participant-clustered Cox models used robust sandwich standard errors clustered by participant ID and were stratified by propensity score–matched subclass. In the one-hip-per-participant sensitivity analysis, one hip was randomly selected per participant before propensity score matching, after which the matching and Cox analyses were repeated. CSA, cross-sectional area; CI, confidence interval; HR, hazard ratio.

**Table S7. Exploratory Data-Derived cutpoints for 2-year CSA changes**

| **Muscle region** | **Z-score-normalized cutpoint** | **Raw CSA change cutpoint, mm²** |
| --- | --- | --- |
| Adductors | −0.92 | −164.09 |
| Quadriceps | −0.03 | −101.43 |
| Hamstrings | −0.57 | −142.15 |
| Total muscle | −0.02 | −161.23 |

Cutpoints were selected and evaluated in the same dataset without bootstrap or external validation. They are exploratory, may be optimistic, and should not be used as clinically validated thresholds.

**Part 3**

**Fig. S1. Covariate Balance before and after propensity score matching.** Absolute standardized mean differences are presented for baseline covariates before and after 1:4 propensity score matching. The dashed vertical line indicates the prespecified balance threshold of 0.10. All covariates had absolute standardized mean differences below 0.10 after matching. SMD, standardized mean difference.
